# Late metastasis to macroscopically normal paranasal sinuses from breast cancer

**DOI:** 10.3332/ecancer.2013.298

**Published:** 2013-03-21

**Authors:** James Johnston, Michael George, Petros D Karkos, Rhagav C Dwivedi, Samuel C Leong

**Affiliations:** 1 Department of Otolaryngology, Queen Alexandra Hospital, Southwick Hill Road, Portsmouth, UK; 2 Department of Otolaryngology, Ahepa Univeristy Hospital, Thessaloniki, Greece; 3 University Hospital Aintree, Liverpool, UK

**Keywords:** breast cancer, metastasis, paranasal sinuses, sphenoid, ethmoid

## Abstract

**Background:**

Breast cancer can very rarely result in late metastases to the paranasal sinuses.

**Methods and results:**

We present a 75-year-old woman who developed sinonasal symptoms mimicking sinusitis 20 years after receiving a breast cancer diagnosis. Rigid nasendoscopy was unremarkable, but due to persistent unilateral nasal symptoms and suspicious radiological findings, the patient underwent endoscopic biopsies of macroscopically normal sinuses which confirmed metastatic breast cancer.

**Conclusions:**

High suspicion index, a thorough history, and examination are of paramount importance as metastases to the sinuses from breast cancer can occur even 20 years after diagnosis.

## Introduction

Breast cancer metastases to the paranasal sinuses are very rare with few cases documented in the literature [1–6]. We present a case of late metastasis to the paranasal sinuses that resulted from breast cancer (initially diagnosed in 1983), and we review the current literature regarding presentation and diagnosis.

## Case report

**Figure 1:**
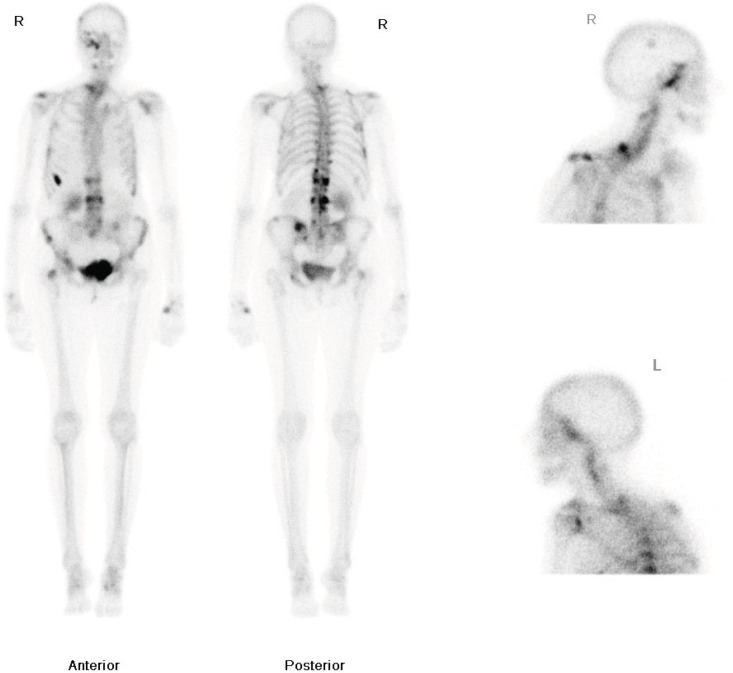
Whole body nuclear medicine bone scan showing increased uptake in the right ethmoid and maxillary sinuses.

A 75-year-old lady with a history of breast carcinoma presented to her general practitioner (GP) with right-sided nasal blockage and pain. These symptoms were associated with right-sided numbness in the distribution of the maxillary division of the trigeminal nerve. She was urgently referred to the Ear, Nose, and Throat (ENT) clinic. The patient had a medical history of a T1cN1Mo oestrogen receptor-positive, right-sided ductal breast carcinoma, diagnosed in 1983. She was treated with a lumpectomy and radiotherapy. She subsequently developed a second left-breast T2NOMO ductal carcinoma in 1991, which was treated surgically by mastectomy. Following her mastectomy, she received adjuvant Tamoxifen for 10 years. In 2009, she developed pain in the thoracic area and a bone scan was requested. This demonstrated increased uptake in the dorsal-thoracic and lumbar spine, left hemipelvis, right femoral neck, ribs, right skullbase, and sphenoethmoidal recess (Figure 1). She was treated with radiotherapy to the lumbar spine (8 Gy single fraction) and started on letrozole due to the oestrogen receptor-positive status of the primary tumour. The patient was started on a course of IV pamidronate, along with monitoring bone scans, to reduce the risk of osteoporosis related to both letrozole therapy, and repeat dual energy X-ray absorptiometry (DEXA). Over the following two years, her repeat scans did not show progression of the disease process and she remained well on letrozole therapy, until she presented to her GP with the symptoms noted above.

**Figure 2:**
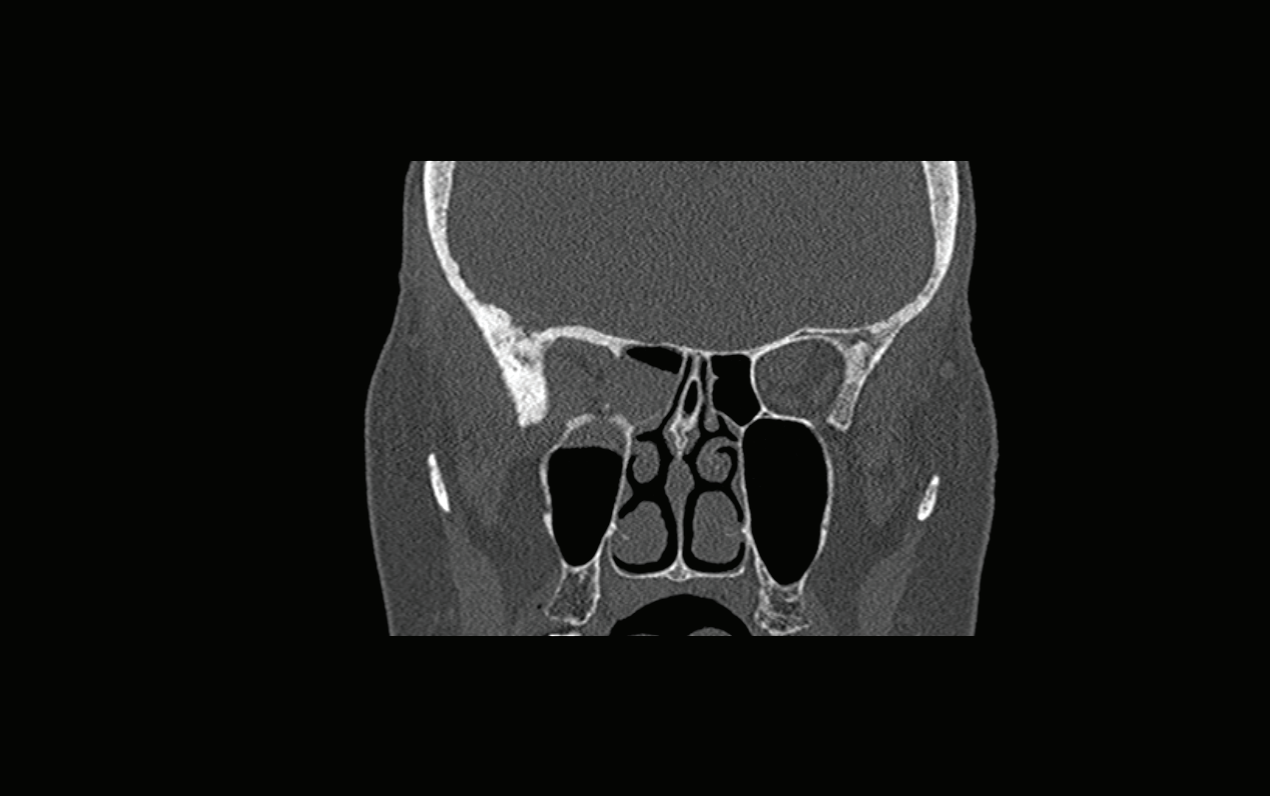
Coronal CT scan showing non-specific findings of “mucosal disease” involving the right maxillary, ethmoid, and sphenoid sinuses. An erosion of the lateral wall of right sphenoid is also noted.

Flexible nasendoscopy was performed in ENT outpatient clinic and did not identify any lesions or mucosal abnormality. Her most recent bone scan revealed an increased uptake in the right ethmoid sinus as well as the other areas noted previously. The outcome is similar to her original scan in 2009 as well as her subsequent scans. Due to her new symptoms, she was investigated further with a computerized tomography (CT) scan of her sinuses. This showed minor right-sided ethmoid sinus involvement and soft tissue density in the right sphenoid and maxillary sinuses (Figure 2).

The case was discussed during the hospital multidisciplinary oncology meeting and a decision to proceed to surgical biopsies was taken. Endoscopic sinus surgery was arranged on an urgent 2-week wait basis and biopsies were taken from the macroscopically “normal” right and left maxillary, ethmoid, and sphenoid sinuses. All three biopsies from the right side showed fragments of respiratory mucosa infiltrated by poorly differentiated adenocarcinoma which were subsequently confirmed as ER and PR positive. These findings were consistent with metastases from her primary breast carcinoma.

Following multi-disciplinary discussion, the patient was advised that letrozole should be discontinued due to the progression of her symptoms whilst on treatment. Capecitabine chemotherapy was planned and the patient was started on exemestane and dexamethasone. A repeat staging CT of the patient’s neck, chest, abdomen, and pelvis showed a shallow pleural effusion, as well as a lung parenchymal lesion in the lingular lobe with a pleural deposit at the left base abutting the aorta and oesophagus. There was also evidence of sclerotic infiltration in the pelvis, proximal femora, sacrum, multiple vertebral bodies, and posterior spinal elements, right humeral head, medial left clavicle, and multiple ribs. Capecitabine therapy was initiated as planned.

## Discussion

Whilst breast cancers have varied sites for metastases including bone, lung, liver, and the brain [1], metastasis from a breast carcinoma to the paranasal sinuses is rare, having less than 20 reported cases in the literature [2]. Metastases to the ethmoid sinuses are rarer still, with only three documented cases from a breast primary [3, 4]. The most common site within the paranasal sinuses for breast cancer metastases is the maxilla/maxillary antrum [3, 5]. Bernstein *et al., *reported that a breast malignancy was the third most common cause for metastasis to the paranasal sinuses; the most common source is a renal primary. This has been confirmed in subsequent reviews of the literature [4, 6].

The common presenting symptoms of paranasal sinus metastasis mimic those of rhinosinusitis and, therefore, can lead to a delay in diagnosis, particularly in the presence of immunosuppressive drugs. In this case, the patient presented to the GP with symptoms of nasal obstruction and discomfort; this was associated with numbness in the distribution of the maxillary division of the trigeminal nerve, so the GP was concerned enough to refer urgently to ENT. She did not describe any epistaxis. Epistaxis is a common feature of renal metastases to the paranasal sinuses of her but very uncommon in breast metastases [5]. On review in clinic, she was not found to have the other features of Trotter’s triad (glue ear and an immobile soft palate). In addition, flexible nasendoscopy did not reveal any mucosal abnormality or mass within the nasal cavities. Due to a high index of suspicion, she underwent a repeat bone scan and subsequent CT sinuses, which revealed abnormalities in the right maxillary, ethmoid, and sphenoid sinuses. Urgent endoscopic surgery biopsies revealed oestrogen and progestogen receptor-positive adenocarcinoma confirming the clinical suspicion of metastatic breast cancer.

Although the patient displayed the symptoms commonly associated with this diagnosis, this presentation is unusual as flexible nasendoscopy did not reveal an obvious mass or mucosal abnormality. In most of the reports in the literature, the patient has a mass lesion within the sinuses on examination at the time of presentation [4, 5, 7]. Also, the bone scan findings were non-specific, as there is a variable degree of uptake in sinusitis as well as metastases. In addition, there had been little radiological progression in her scans over a two year period. For these reasons, a high index of suspicion was required to obtain the correct diagnosis.

Breast carcinoma can metastasise by both haematogenous and lymphatic routes. Nahum and Bailey suggested that the most likely route was haematogenous, via the vena cava and pulmonary circulation; and subsequently to the sinuses, via the arterial supply of the head and neck [8]. However, they also cite the work of Batson on the vertebral veins and jugular venous system where he demonstrated retrograde flow on valsalva manoeuvre; which was sufficient to reach the skull-base at times, as a possible explanation [9]. Batson’s work has been accepted as the most likely explanation for these metastases in subsequent papers [3–5, 7].

## Conclusion

Metastases to the paranasal sinuses from breast cancer are rare. The symptoms often mimic sinusitis, and unlike renal metastases patients do not present with epistaxis. In addition, although there are usually signs such as mucosal abnormality or an intranasal mass on examination, these may be absent as in the case we describe. This, along with the fact that patients may present 20 years or more after their initial diagnosis, means that a thorough history and examination coupled with a high index of suspicion are required. Imaging in the form of high-resolution CT or magnetic resonance imaging (MRI) can help to delineate involved areas. Tissue biopsies even from macroscopically “normal” sinuses may be necessary to reach the correct diagnosis.

**Financial disclosure:** none

**Conflict of Interest:** none

## References

[1] Carty NJ, Foggitt A, Hamilton CR, Royle GT, Taylor I (1995). Patterns of clinical metastasis in breast cancer: an analysis of 100 patients. Eur J Surg Oncol.

[2] Prescher A, Brors D (2001). Metastases to the paranasal sinuses: case report and review of the literature. Laryngorhinootologie.

[3] Austin JR, Kershiznek MM, McGill D, Austin SG (1995). Breast carcinoma metastatic to paranasal sinuses. Head Neck.

[4] Fyrmpas G, Adeniyi A, Baer S (2011). Occult renal cell carcinoma manifesting with epistaxis in a woman: a case report. J Med Case Rep.

[5] Bernstein JM, Montgomery WW, Balogh K (1966). Metastatic tumors to the maxilla, nose and paranasal sinuses. Laryngoscope.

[6] Simo R, Sykes AJ, Hargreaves SP, Axon PR, Birzgalis AR, Slevin NJ (2000). Metastatic renal cell carcinoma to the nose and paranasal sinuses. Head Neck.

[7] Davey S, Baer S (2012). A rare case of breast cancer metastasising to the nasopharynx and paranasal sinuses. Int J Surg Case Rep.

[8] Nahum AM, Bailey BJ (1963). Malignant tumors metastatic to the paranasal sinuses. Laryngoscope.

[9] Batson OV (1940). The function of the vertebral veins and their role in the spread of metastases. Ann Surg.

